# Natural Polyphenols for Treatment of Colorectal Cancer

**DOI:** 10.3390/molecules27248810

**Published:** 2022-12-12

**Authors:** Yiwen Zhang, Kunjian Liu, Chengqiu Yan, Yu Yin, Shuangyan He, Li Qiu, Guofeng Li

**Affiliations:** 1 College of Chinese Medicine, Changchun University of Chinese Medicine, Changchun 130117, China; 2 Anorectal Department, Affiliated Hospital of Changchun University of Traditional Chinese Medicine, Changchun 130021, China

**Keywords:** colorectal cancer, polyphenol, chemoprevention, combination therapy

## Abstract

Colorectal cancer (CRC) is a prevalent and serious gastrointestinal malignancy with high mortality and morbidity. Chemoprevention refers to a newly emerged strategy that uses drugs with chemopreventive properties to promote antioxidation, regulate cancer cell cycle, suppress proliferation, and induce cellular apoptosis, so as to improve cancer treatment outcomes. Natural polyphenols are currently recognized as a class of chemopreventive agents that have shown remarkable anticarcinogenic properties. Numerous in vitro and in vivo studies have elucidated the anti-CRC mechanisms of natural polyphenols, such as regulation of various molecular and signaling pathways. Natural polyphenols are also reportedly capable of modulating the gut microbiota and cancer stem cells (CSCs) to suppress tumor formation and progression. Combined use of different natural polyphenols is recommended due to their low bioavailability and instability, and combination treatment can exert synergistical effects, reduce side effects, and avoid drug resistance in CRC treatment. In summary, the application of polyphenols in the chemoprevention and treatment of CRC is promising. Further clinical evaluation of their effectiveness is warranted and anticipated.

## 1. Introduction

Colorectal cancer (CRC) is a prevalent gastrointestinal malignancy and the second leading cause of death in cancer patients [1]. The global incidence of CRC ranks third among all types of cancers and continues to rise [1]. The pathogenesis of CRC is multifactorial, involving genetic factors, dietary habits, lifestyle (such as excessive intake of processed meat, drinking, and smoking), and excess body weight [2]. Despite the current progress in CRC treatment, the 5-year survival of patients remains poor, which indicates the limitations of conventional treatments, including surgery, chemotherapy, radiotherapy, and immunotherapy, and highlights the need for new therapeutic approaches against CRC.

**Figure 1:**
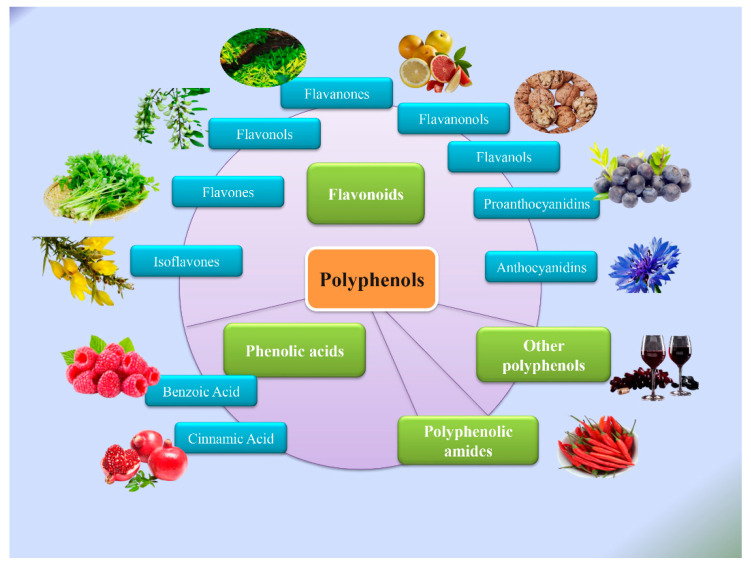
Classification of polyphenols.

Natural products originating from plants have recently attracted considerable attention due to their potential benefits as chemotherapeutic and prophylactic agents against cancers [3]. Multiple natural polyphenols have been identified, including flavonoids, phenolic acids, polyphenolic amides, and other polyphenols. Flavonoids and phenolic acids account for nearly 60% and 30%, respectively, of the currently known polyphenols [4]. The efficacy of other important polyphenols, such as curcumin and resveratrol, has also been demonstrated in the treatment of various diseases (Figure 1).

**Figure 2:**
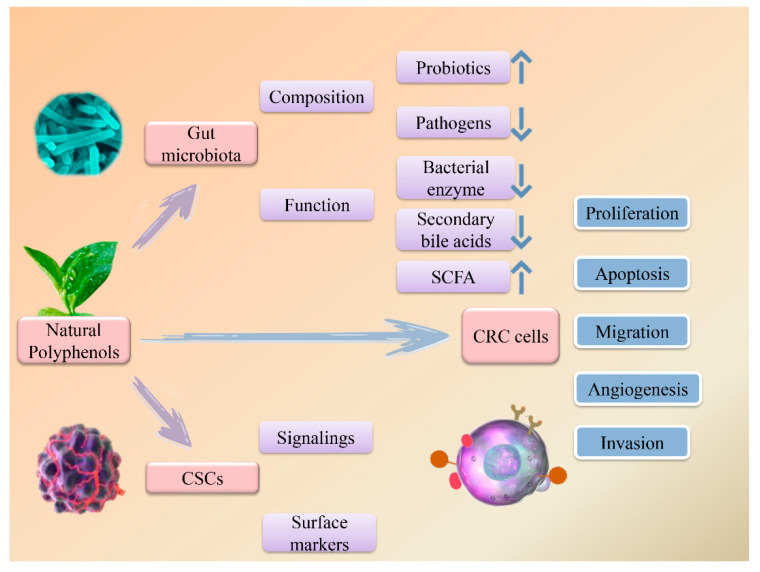
The role of polyphenols in the gut microbiota, cancer stem cells (CSCs), and colorectal cancer cells (CRC).

The mechanisms underlying the therapeutic and preventive effects of natural polyphenols against CRC are associated with regulation of various molecules and signaling pathways, inhibition of cancer cell proliferation, migration, and invasion, and promotion of apoptosis. These agents also exert anti-cancer effects via regulating the gut microbiota and cancer stem cells (CSCs), which are considered crucial in the pathogenesis of CRC (Figure 2). Therefore, we have reviewed studies regarding natural polyphenols for CRC treatment, summarized the regulatory effects of polyphenols on the gut microbiota and CSCs in CRC patients, and provided an overview of the potential mechanisms of several commonly used polyphenols. We have also outlined the synergistic anti-cancer effects of combination polyphenol therapy. We expect this paper to provide direction and serve as a reference for the development of effective anti-CRC agents to improve the survival of patients.

## 2. Polyphenols for Gut Microbiota Regulation

It is well recognized that a homeostatic gut microbiota is essential for the overall health of the host. A dynamic balance is maintained among various kinds of intestinal bacteria, which is delicate and can be easily broken by intestinal carcinogenesis, leading to dysbacteriosis. A possible model of the association between the microbiota and CRC showed that certain intestinal commensal bacteria may cause DNA damage and gene mutations in intestinal epithelial cells, leading to alterations in the intestinal microenvironment and the proliferation of certain opportunistic pathogens [5]. The expansion of pathogenic microbiota also contributes to cancer progression. For example, *Fusobacterium nucleatum* induces oncogenic processes by inducing DNA damage, reactive oxygen species (ROS) generation, and CRC cell growth [6]. *Enterococcus faecalis* produces extracellular free radicals that increase chromosomal instability associated with CRC [7]. Increased DNA damage has been reported in colonic luminal cells of rats colonized with *Enterococcus faecalis*. Some other gut microbes, such as *Escherichia coli*, *Bacteroides fragilis*, *Clostridium septicum*, and *Streptococcus bovis*, have also been demonstrated to be associated with CRC genesis and progress [8]. A protective microbiota favors the suppression of CRC. For instance, *Lactobacillus casei* BL23 administration reduced proliferative index values and histological scores in azoxymethane (AOM)-induced CRC mice through its antiproliferative and immunomodulatory effects, thereby inhibiting CRC development [9]. In addition, this probiotic strain appeared to counteract dysbiosis in CRC. Therefore, regulation of the gut microbiota and maintenance of a homeostatic intestinal microenvironment would be a promising approach for CRC treatment.

Natural polyphenols exert chemopreventive effects by improving microbiota metabolism, alleviating intestinal inflammation, and reducing pathological invasion. Polyphenols extracted from green tea have been observed to alter the composition of the gut microbiota in rats. The abundance of Peptostreptococcaceae associated with the CRC phenotype was decreased, while that of *Bacteroidetes* and *Oscillospira* associated with a lean phenotype was increased [10]. Li et al. found an increasing proportion of *Bifidobacterium* and decreased abundance of colitis-associated pathobionts after administration of resveratrol in colitis model mice [11]. Resveratrol ameliorated gut microbiota dysbiosis and restored microbial community diversity by downregulating the expression of pro-inflammatory cytokines. Decreased abundances of the genera *Enterococcus* and *Lactobacillus* were also observed in rats fed polyphenol-rich blueberries [12], and the expression of genes associated with epithelial bacterial invasion were reduced 8-fold in the blueberry-treated group.

Polyphenols regulate the intestinal microbiota by promoting colonization of probiotics while suppressing opportunistic pathogens, thus restoring intestinal microbial balance [13,14]. Administration of epigallocatechin-3-gallate (EGCG) resulted in a decreased number of precancerous lesions and reduced solid tumor size in mice with CRC induced with AOM/dextran sodium sulfate (DSS) [15]. The abundances of probiotic Bifidobacteria and *Lactobacillus* were enriched while the diversity of the gut microbiota remained relatively stable in mice administered EGCG. Increased gut microbial diversity in AOM/DSS-induced CRC model mice was reported, with decreased proportion of butyrate-producing bacteria and increased abundance of potential oncogenic bacteria, such as *Bacteroides*. Similarly, in an AOM/DSS-induced mouse model, Chen et al. observed that the abundances of pathogenic bacteria, such as *Desulfovibrio* spp. and *Enterococcus* spp., were inhibited, while those of probiotic bacteria were enhanced in the group receiving black raspberry anthocyanins [16]. The authors further revealed that anthocyanins could mediate SFRP2 demethylation and alleviate inflammation. Intestinal dysbacteriosis is probably an early event in cancer progression, leading to epigenetic changes and inflammatory response. In AOM-induced interleukin (IL)-10-deficient mice, curcumin administration resulted in increased numbers of *Lactobacilli* colonies and microbiota richness, with subsequently alleviated tumor burden, suggesting a correlation between the microbiota and reduced tumor burden [17]. High microbial similarity was observed between curcumin-administered CRC model mice and those in the control group, indicating that curcumin may be effective in counterbalancing disease-induced dysbiosis.

Polyphenols can also regulate the gut microbiota by intervening in microbial metabolism. Molan et al. reported significantly decreased activity of β-glucuronidase, a known bacterial enzyme associated with CRC, in rats fed green tea extract (GTE) by gavage [18]. Dietary polyphenols in rats administered a high-fat diet induced a significant decrease in levels of fecal secondary bile acids [19], which were closely associated with increased risk for CRC [20]. A randomized controlled trial that investigated the effects of cranberry polyphenols on the gut microbiota recruited 11 healthy adults to be given an animal-based diet [21]. These participants had increased levels of secondary bile acids with decreased abundance of short-chain fatty acids (SCFAs), which was reversed by cranberry polyphenol treatment. The potential mechanism might involve regulation of the gut microbiota.

Gut microbiota-produced polyphenol metabolites also exert anticancer effects against CRC, which may further increase the overall efficacy of polyphenols. A study compared the cytotoxicity of quercetin and its metabolites across colon cancer cell lines [22]. The metabolites produced from some gut microbiota, such as *Clostridium perfringens* and *Bacteroides fragilis,* strongly inhibited specific cell lines relative to quercetin. Resveratrol metabolites inherited anticancer properties from their parent, which were manifested as proliferation suppression, cell cycle blocking, and apoptosis activation in CRC cells [23]. A mixture of ellagitannin metabolites reduced the size and number of colonospheres in colonic CSCs, accompanied with decreased molecular and phenotypic CSC characteristics [24].

## 3. Polyphenols modulate CSCs

CSCs are capable of self-renewal and differentiation, and have further regenerative abilities for carcinogenesis and metastasis [25]. CSCs are usually present in a quiescent cell cycle stage and are insensitive to radiotherapeutic and chemotherapeutic agents. CSC invasion is an important cause of tumor treatment failure. The markers of colonic CSCs are characterized by tumorigenicity and acquaintance of metastatic potential, which mainly includes CD133, CD44, CD29, CD24, CD166, Aldh1, EpCAM, ESA, and LGR5 [26]. These markers are effective indicators for discriminating CSC viability. Therefore, CSCs present a major challenge for overcoming tumorigenesis, drug resistance, tumor recurrence, and metastasis. Signaling pathways or surface markers of CSCs would be promising targets for CRC treatment, for which polyphenols would be potentially competent.

A novel curcumin analog, GO-Y030, decreased cell viability, inhibited tumorsphere formation, and promoted apoptosis in colonic CSCs via downregulation of the STAT3 pathway [27]. Tumor volume was also inhibited by GO-Y030 in mice with xenografted CSCs. Difluorinated curcumin (CDF), another analog of curcumin, reduced levels of CSC markers, suppressed proliferation, and disintegrated the colonospheres in chemo-resistant CRC cells [28]. These chemo-resistant cells were enriched in CSCs. Roy et al. revealed that the inhibition of CDF on CSCs was mediated by the miRNA-21-PTEN-Akt axis [29]. CDF downregulated miRNA-21 and upregulated the PTEN pathway, resulting in reduced expression of Akt. Toden et al. showed that the Notch1 pathway was downregulated, while self-renewal suppressive-miRNAs were upregulated by EGCG, leading to suppressed proliferation and attenuated chemoresistance of CSCs [30]. Additionally, EGCG was able to suppress cell growth and the spheroid formation ability of colonic CSCs, which were attributed to inhibition of the Wnt/β-catenin pathway [31]. The latter was also involved in resveratrol-induced colonic CSC apoptosis [32]. Grape-extracted resveratrol resulted in mitochondrial-mediated and TP53-independent cell death by suppressing the downstream genes associated with the Wnt/β-catenin pathway [32]. Another study reported decreased CSC resistance to chemotherapeutic agents and their clonogenic capacities after resveratrol administration, which was accompanied by enhanced autophagy signaling and GALNT11 suppression [33]. GALNT11 expression is typically associated with malignancies and tumor recurrence. The above findings indicate that polyphenols, by regulating different pathways in colonic CSCs, have favorable therapeutic potential against CRC.

## 4. Typical Polyphenols and Their Anti-CRC Effects

Typical polyphenols, their anti-CRC effects, and related mechanisms are summarized in Table 1.

### 4.1. Curcumin 

**Figure 3:**
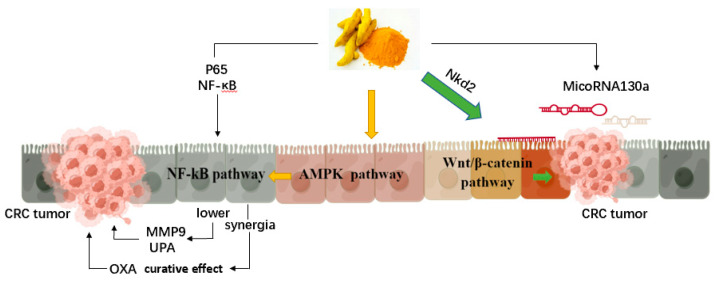
Mechanism of action of curcumin on colorectal cancer cells. Created by Figdraw.

Curcumin is a bioactive monomer isolated from *Curcuma longa*. It has been demonstrated to be a phytochemical agent with anti-proliferative, anti-inflammatory, and antioxidative properties. Curcumin is effective against various cancers, especially CRC (Figure 3). Multiple signaling pathways are involved in CRC genesis, among which the Wnt cascade is considered to play a crucial role. Gene mutations in Wnt signaling players, such as β-catenin and APC, are found in most CRC patients [72]. Curcumin suppressed cell proliferation in the SW480 cell line, which was attributed to inhibition of the Wnt/β-catenin pathway and downregulation of miRNA-130a [34]. Regulation of miRNAs also contributes to the anti-CRC activity of curcumin. Overexpression of miRNA-130a led to the recovery of curcumin-suppressed CRC cells [34]. Tong et al. found that curcumin could inhibit the proliferation and adhesion abilities of colonic cancer cells [35]. Matrix metalloproteinase-9 (MMP9) and urokinase-type plasminogen activator (uPA) play a regulatory role in cancer cell invasion, which would be associated with AMPK activation and NF-κB inhibition. Curcumin made drug-resistant CRC cells more responsive to oxaliplatin (OXA) via downregulation of NF-κB expression [73]. Curcumin combined with OXA caused massive cell death with less colony formation compared to curcumin or OXA monotreatment. Curcumin administration resulted in inhibited tumor growth in rats with liver metastases derived from CRC cells [36]. Interestingly, curcumin can affect the distribution of tumors in different locations of liver lobes, and the underlying reason warrants further investigation. Furthermore, the results of clinical trials have revealed that oral administration of curcumin in combination with chemotherapy may be well tolerated and safe for metastatic CRC patients [74,75].

### 4.2. Resveratrol

Resveratrol, which is isolated from mulberries, peanuts, and grapes, has potentially remarkable anti-CRC properties involving multiple signaling pathways, such as Wnt, NF-κB, AMPK, ROS, and caspases, among others [76]. For instance, a study indicated that resveratrol was pro-apoptotic and decreased cellular viability in CRC cell lines by promoting ROS generation and activating the mitochondrial-dependent apoptotic pathway [37]. The MAPK pathway was also involved in activating cellular apoptosis induced by resveratrol, possibly owing to upregulated expression of bone morphogenetic protein 9 (BMP9) [38]. Resveratrol also regulated Wnt/β-catenin signaling and its downstream gene, MALAT1, thereby inhibiting CRC cell invasion and metastasis [39]. In an in vivo CRC lung metastasis model, Yuan et al. found fewer lung metastases in mice after treatment with resveratrol [40]. Histopathological staining also showed that the group treated with resveratrol exhibited decreased tumor vascularization and tumor cell density. The authors speculated that suppression of CRC metastasis after resveratrol administration might be associated with regulation of the AKT/GSK-3β/Snail pathway and epithelial mesenchymal transition (EMT). Furthermore, a clinical trial investigated the effect of oral resveratrol administration before surgery on patients with CRC [77]. The results showed an additional decrease in Ki-67 levels in tumor tissues following resveratrol administration, indicating the suppression of cancer cell proliferation. Another trial revealed that micronized resveratrol induced the expression of several pro-apoptotic proteins, such as cleaved caspase-3, in malignant liver tissues from patients with CRC hepatic metastases [78]. The trial also confirmed the safety and tolerability of resveratrol.

### 4.3. EGCG

Green tea contains several biologically active polyphenols, from which EGCG is extracted and exerts an antagonizing effect against CRC. [41]. EGCG is a subgroup of flavanols classified as flavonoids. EGCG inhibits the expression of vascular endothelial growth factor (VEGF) and hypoxia-inducible factor-1alpha (HIF-1α), both of which promote tumoral angiogenesis, thus suppressing CRC cell growth [42]. EGCG activated the apoptotic cascade in colonic cancer cells to decrease the tumor size of xenografts via downregulation of the PI3K/AKT pathway [79]. Other studies have revealed that AMPK, Notch, and MAPK are promising targeted pathways in regulating the proliferation and apoptosis of CRC cells [80,81,82]. In another study using a dimethylhydrazine (DMH)-induced CRC model, the tumor formation rate, number, and volume were reduced after EGCG treatment [43]. In detail, the results showed that precancerous lesion and ascite formation in rats was suppressed by EGCG. Moreover, a clinical trial reported that the administration of GTE (mainly EGCG) reduced the expression of DNA methyltransferase (DNMT1) and NF-κB mRNA in human rectal tissue, indicating a regulatory role for EGCG in CRC tumorigenesis-related biomarkers [83]. However, another clinical trial revealed no significant differences in the formation of new precancerous lesions between the EGCG intervention and control groups [84]. Prior to EGCG intervention, patients were identified by chromoendoscopy with at least five rectal malformation crypt foci (ACF) or precursor lesions to CRC. A current trial involving 632 patients with histories of colonic adenoma has found a slight decrease in the recurrence rate of adenoma in patients given GTE compared to the control group [85]. Interestingly, male patients receiving oral GTE showed a lower rate of adenoma formation than those receiving a placebo. Although EGCG is well tolerated, more trials are required to demonstrate its additional benefits in human subjects. 

### 4.4. Quercetin

Quercetin is a flavonol compound in the flavonoid family that is derived from onions, asparagus, and berries. Quercetin shows anti-CRC effects through the regulation of different molecular mechanisms. Kee et al. revealed that the pro-apoptotic effect of quercetin could be due to the activation of the MAPK signaling pathway in colonic cancer cells and reduced cell migration through suppression of MMP-2 and MMP-9 [44]. MMP is a promoter of tumor metastasis following ECM cleavage. Quercetin also inhibited colorectal cell lung metastasis in a mouse model, in which a decreased number of tumor nodules and lessened lung lesions were observed. This cytotoxic effect of quercetin on CRC cells was explained by its ability to induce ROS generation via COX-2 [45]. COX-2 is usually overexpressed during tumorigenesis in CRC. Downregulation of the Wnt/β-catenin pathway and related genes (cyclin D1 and survivin) was involved in quercetin-induced apoptotic episodes in colonic cancer cells [46]. Other pathways, such as PI3K/AKT/mTOR, JNK/JUN, and NF-κB, could also be regulated by quercetin, thus exerting an inhibitive effect against CRC cells [86,87]. An in vivo study in mice showed that quercetin exerted anti-CRC effects through its anti-inflammation and antioxidant properties, resulting in reduced tumor size, attenuated inflammation, and downregulation of oxidative stress markers [47]. A clinical trial was conducted to evaluate the effectiveness of curcumin and quercetin for patients with familial adenomatous polyposis after prior surgical resection [88]. They recruited 5 patients, all of whom exhibited a reduced number of polyps and tumor size. Similarly, another trial found that high intake of flavonol-containing quercetin caused a decrease in the risk for adenoma recurrence [89]. A meta-analysis of 12 studies reported that high flavonol and flavone intake might lead to decreased risk for colorectal carcinogenesis [90]. 

### 4.5. Apigenin and Luteolin

**Figure 4:**
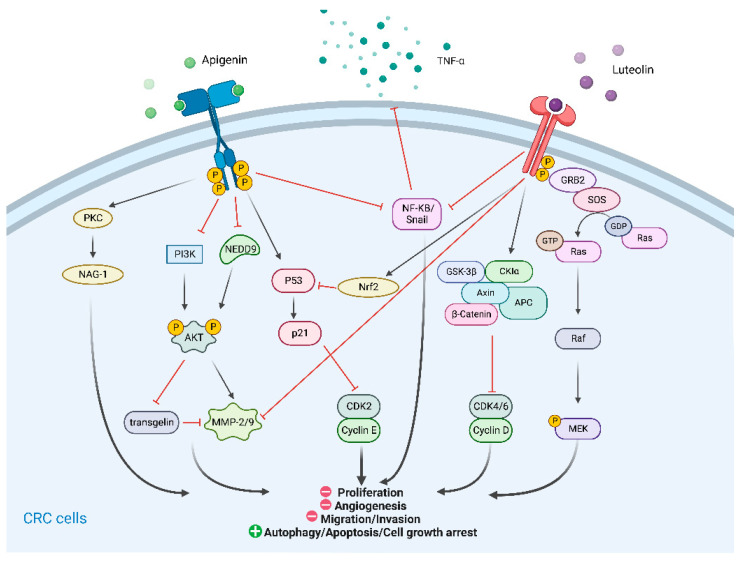
Mechanism of apigenin and luteolin against colorectal cancer cells. Created by Bio render.

Both apigenin and luteolin are flavones, a type of flavonoid. They show some similarities in their activities against CRC (Figure 4).

Apigenin has been shown to inhibit the proliferation and migration in CRC cells via upregulation of transgelin expression and downregulation of MMP-9 expression, mediated through the Akt pathway [48]. Transgelin is a repressor of MMP-9. Another study reported that apigenin could decrease NEDD9 expression, leading to inhibition of the Akt pathway and subsequent migration and invasion of CRC cells [49]. NEDD9 acts as a vital regulator in cancer progression and metastasis. Apigenin also inhibited EMT to intervene in CRC cell migration through the NF-κB/Snail pathway [91]. In cisplatin-resistant colon cancer cells, autophagy and apoptosis were induced by apigenin treatment through the suppression of the mTOR/PI3K/AKT pathway [92]. mTOR/AKT signaling was also implicated in apigenin-induced downregulation of Wnt/β-catenin, leading to decreased cell survival [93]. Moreover, apigenin enhanced cell growth arrest and apoptosis, accompanied by increased expression of p21 and NAG-1 [50]. These proteins are activated by the p53 and PKC pathways. Polyp numbers were reduced in a APC^Min/+^ (Min, multiple intestinal neoplasias) mice model, and p53 expression was increased in these tumor tissues after apigenin treatment. APC^Min/+^ mice are characterized by a phenotype with multiple intestinal neoplasia and mutations in the APC gene. The anti-CRC activities of apigenin have also been achieved by suppressing tumorigenesis through the repression of HIF-1 and VEGF in tumor tissues [51]. A prospective cohort trial evaluating the effects of a flavonoid mixture (apigenin and EGCG) on tumor recurrence enrolled 87 patients after colectomy or polypectomy, among whom 46 patients eventually underwent colonoscopic surveillance. The results showed that patients supplemented with flavonoids had a significantly lower risk of tumor recurrence compared to those without supplementation [94].

Luteolin has been shown to sensitize tumor necrosis factor (TNF)-alpha, thereby inducing apoptosis in CRC cells through the suppression of NF-κB and its targeted genes [52]. Pandurangan et al. found that cell cycle arrest and apoptosis were promoted by luteolin in colon cancer cells, which was regulated by glycogen synthase kinase-3β and cyclin D1 through the Wnt/β-catenin pathway [53]. In an AOM-induced CRC mouse model, they demonstrated other mechanisms underlying the anti-CRC properties of luteolin. Luteolin affected the cell membrane glycoprotein by exerting an antioxidant effect, thereby reducing pre-neoplastic lesions and suppressing tumor development [95]. The expression of metastatic tumor markers MMP-9 and MMP-2 was suppressed in mice following luteolin supplementation, indicating that luteolin possessed anti-metastatic properties against CRC [54]. Treatment with luteolin led to activation of the Nrf2 pathway, which diminished tumor progression [96]. A previous study indicated a significantly higher incidence of CRC adenocarcinoma in AOM-treated Nrf2-knockout mice than in wild-type one, which suggested the importance of Nrf2 in CRC genesis [97]. Another study suggested that the Nrf2-activated anti-apoptosis effect of luteolin on colon cells was realized through DNA demethylation induction [55]. An interaction exists between the Nrf2 and p53 pathways, which could upregulate the expression of pro-apoptotic proteins and antioxidant enzymes [55]. Moreover, the p53 pathway is also reportedly involved in luteolin-induced cell cycle arrest and cellular apoptosis in colonic cancer cells [98]. On the other hand, Kang et al. discovered that luteolin exerted its anti-apoptosis effect by enhancing antioxidant activity and upregulating the MAPK pathway [99]. Other pathways or target molecules, such as Raf and PI3K/Akt [100], insulin-like growth factor-I receptor [101], ERK/FOXO3a [102], and miRNA-384/pleiotrophin [103], have been associated with the repression of CRC by luteolin.

### 4.6. Anthocyanins

Anthocyanins exist abundantly in various vegetables, fruits, and wine, and are key components of the flavonoid family. A recently published meta-analysis of 7 observational studies showed that anthocyanin administration contributes to a lower risk of CRC genesis [104]. Shin et al. found that anthocyanins extracted from grapes could increase the expression of pro-apoptotic proteins, such as caspase-3, -8, and -9, while decreasing that of anti-apoptotic proteins, leading to cell death in HCT-116 cells [56]. Grape anthocyanins modulated tight junction proteins (TJs) and decreased the expression of MMPs to attenuate cellular migration [57]. The tightness of TJs is important for maintaining cell–cell adhesion. In the above two studies, both the pro-apoptotic and anti-migration activities exerted by grape anthocyanins are due to p38-MAPK upregulation and Akt downregulation. A study by Anwar et al. demonstrated that a berry anthocyanin-rich extract was capable of decreasing CRC cell viability by suppressing cyclin-dependent kinase inhibitor 1 (p21Waf/Cif1), activating caspase-3, and enhancing ROS production [58]. *Aronia melanocarpa* Elliot anthocyanins inhibited Wnt/β-catenin activity in Caco-2 cells, resulting in inhibited cell growth and enhanced apoptosis induction [105]. Anthocyanins derived from purple-fleshed potatoes inhibited cell growth and sphere formation of colonic CSCs, which were also regulated by the Wnt/β-catenin pathway [106]. Black raspberries containing abundant anthocyanins exerted a demethylation effect via the Wnt pathway, which demethylated the upstream regulators of Wnt to downregulate the expression of DNA methyltransferase enzymes DNMT1 and DNMT3B [107]. Strawberries containing abundant anthocyanins inhibited the incidence of inflammation, which promoted colon tumors in mice, through the repression of proinflammatory mediators and oncogenic pathways, including PI3K, Akt, ERK, and NF-κB [59]. In addition, Asadi et al. found fewer adenomas in APC^Min^ mice fed sweet potatoes containing high levels of anthocyanins [108].

### 4.7. Gallic Acid

Gallic acid refers to a subclass of phenolic acids that can be isolated from various plants and foods. Its anti-CRC properties can probably be attributed to its antioxidant activity. Gallic acid induced cell cycle arrest, apoptosis, and colony formation inhibition in the HCT-15 cell line, which depended on the ROS-mitochondrial pathway [60]. The extract of pistachio green hulls, mainly containing gallic acid and catechin hydrate, blocked the cell cycle, activated apoptosis, and caused DNA damage in HT-29 cell lines through oxidative pathways [109]. Sanchez-Martin et al. provided another possible mechanism by which gallic acid exerted anti-CRC activity [61], whereby it could bind to DNA G-quadruplexes (G4s) to inhibit cell proliferation and increase DNA damage in SW480 cells. G4s serve as regulators for the transcription, duplication, and genome stability of genes that are associated with tumorigenesis [110]. Tumor volume in xenograft mice and Ki67 expression in tumor tissues were reduced following treatment with gallic acid [61]. Moreover, Giftson et al. showed that DMH-induced CRC model rats administered gallic acid had enhanced lipid peroxidation and antioxidant expression, thus suppressing colon carcinogenesis [62]. Another study revealed that supplementation with gallic acid could suppress the activity of phase I enzymes while enhancing that of phase II enzymes in DMH-induced CRC model rats, resulting in reduced tumor incidence [63].

### 4.8. Capsaicin

Capsaicin is a subgroup of polyphenolic amides that belongs to the polyphenol family. It is a primary pungent constituent found in chili peppers and has been demonstrated as a chemopreventive agent against CRC. Yang et al. speculated that capsaicin could be pro-apoptotic for colonic Colo320DM and LoVo cells via promotion of ROS generation and caspase-3 activation [64]. Similarly, ROS-induced apoptosis was also found in colon 205 cells, along with upregulated expression of caspase-8, -9, and -3 [111]. In addition, the pro-apoptotic effect was also related to AMPK activation [65]. Capsaicin suppressed the expression of β-catenin and intervened in its binding to T-cell factor (TCF), thereby reducing cell proliferation [66]. NAG-1 was identified as another target of capsaicin for apoptosis induction, mediated by C/EBPbeta phosphorylation through the GSK3beta cascade [112]. Moreover, the number and multiplicity of ACF was reduced in CRC rats induced by DMH, showing the preventive effect of capsaicin [67]. However, a low concentration of capsaicin promoted cell invasion and migration in different CRC cell lines (SW480, HCT116, and CT-26) [113]. EMT induction and MMP expression were enhanced by capsaicin via activation of the Akt/mTOR and STAT-3 pathways. It was reported in a recent study that cold and low-dose capsaicin exposure caused ECM remodeling and MMP activation, thus increasing the incidence of tumor cell invasion in DMH-induced CRC model rats [114]. Taken together, further studies evaluating safe concentrations of capsaicin against CRC are needed.

### 4.9. Polyphenols of Other Types

Polyphenols exist abundantly in fruits, vegetables, beans, chocolates, and some sorts of soft drinks, oil grease, and spices. There are many other natural polyphenols in addition to those mentioned above that have been studied in recent years, such as coffee polyphenols. The study by Villota et al. [68] indicated that coffee polyphenols downregulated the expression of CTNNB1, CDH1, and CCND1 and regulated the Wnt/β-catenin pathway, while inhibiting activation of the TCF4 promoter to effectively lower the activity of SW480 cells and suppress migration and invasion. Ferulic acid is a kind of phenolic acid that prevailingly exists in plants. Polymeric and lipidic nanocapsules of ferulic acid inhibited angiogenesis and promoted apoptosis of CRC cells by downregulating cyclin D1, IGF-II, and VEGF and regulating Bax/Bcl-2 [69]. The polyphenol-rich extract from evening primrose seeds (EPE) has also been demonstrated to be capable of inhibiting the activity and migration of colitis-related CRC cells. EPE lowered the invasiveness of CRC cells by downregulating thymidylate synthetase (tyms). Interestingly, in vivo and in vitro studies have found that EPE can lower the invasiveness of 5-FU-resistant CRC cells [70]. Kaempferol is a flavonoid compound that downregulated Ki67 and G-protein-coupled re-ceptor-5 (LGR5) and regulated bile acid secretion to alleviate tumor burden and restore the intestinal mucosal barrier of CRC patients [71].

## 5. Combination Therapy for CRC

### 5.1. Combination of Different Phytochemicals

**Figure 5:**
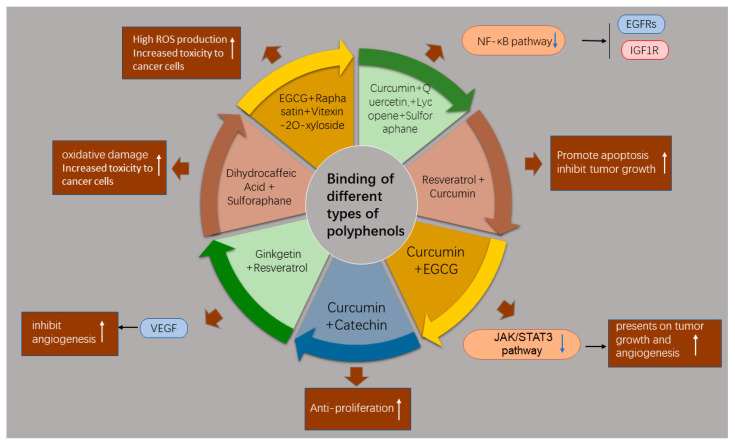
The mechanism diagram of the enhancement of anti-tumor efficacy of combined multi-type polyphenols. White arrows show enhanced efficacy and blue clippings show downregulation.

Different types of polyphenols show anti-CRC activities by targeting various pathways. Recent studies have suggested that two or more polyphenols or their combination with other phytochemicals may produce synergistic anticancer effects by affecting several targets, thus achieving better therapeutic efficacy (Figure 5). This may also allow the use of lower drug doses and diminish the possibility of drug resistance.

It has been demonstrated that the combination of resveratrol and curcumin suppressed HCT-116 cell proliferation and xenograft tumor growth in mice [115], which was attributed to downregulation of the NF-κB pathway and inhibition of EGFRs and IGF-1R [115]. The combination regimen for CRC proved to be more effective than monotherapy with each agent. The curcumin–catechin combination presented higher pro-apoptotic and inhibitive effects on colonic cancer cells than curcumin or catechin monotreatment [116]. Jin et al. established a conditioned medium (CM) for CRC cells to mimic the tumor microenvironment that enhanced tumor angiogenesis [117]. The curcumin–EGCG combination inhibited angiogenesis in CM-induced tumor cells through suppression of the JAK/STAT3 pathway, and the inhibitory effects on tumor growth and angiogenesis were evidently greater in a xenograft mouse model than those observed after EGCG or curcumin monotreatment. Both ginkgetin and resveratrol can target VEGF to affect angiogenesis. The combination of these two agents inhibited the proliferation and tube formation of endothelial cells and subsequent tumor growth in mice, which might have been related to the repression of angiogenesis [118]. Santana-Gálvez et al. investigated the cytotoxicity of dihydrocaffeic acid combined with sulforaphane or curcumin in normal colonic cells and colonic cancer cells [119], reporting that a 1:1 combination of dihydrocaffeic acid and sulforaphane exhibited more cytotoxicity in cancer cells than in healthy cells. The effects were attributed to high oxidative damage caused by dihydrocaffeic acid and sulforaphane. Another study showed that a mixture of EGCG, raphasatin, and vitexin-2-O-xyloside induced high ROS production, leading to increased cytotoxicity in colon cancer cells [120]. Furthermore, Langner et al. used a mixture of curcumin, quercetin, lycopene, and sulforaphane to assess the collective anti-proliferation impact on colon cancer cells [121]. The combination led to enhanced cytotoxic activity in cancer cells while causing no damage to normal epithelial cells.

### 5.2. Combination Treatment with Chemotherapeutic Drugs

Although chemotherapy is crucial for CRC treatment, the adverse effects and potential risk for multidrug resistance have raised concerns, which may be addressed by combination treatment with polyphenols.

5-fluorouracil (5-FU) is one of the most commonly applied agents in CRC chemotherapy. Its use in combination with resveratrol has shown considerable synergistic effects in apoptosis promotion, cell cycle arrest, and EMT repression in CRC cells, and the potential mechanism might involve downregulation of the STAT3/Akt pathway [122]. The pro-apoptotic and anti-telomeric potentials of 5-FU were promoted by resveratrol, thereby re-sensitizing cancer cells to chemotherapy [122]. Curcumin potentiated the cytotoxicity of 5-FU, and the combination of these two agents induced apoptosis to a greater extent in 5-FU-resistant colonic cancer cells [123]. The effects were attributed to the downregulated expression of heat shock protein 27 (HSP-27) and p-glycoprotein (p-gp). EGCG was also capable of increasing the sensitivity of colonic cancer cells to 5-FU cytotoxicity via activation of NF-κB and miRNA-155-5p, thus promoting DNA damage and cell death [124]. The increased expression of miRNA-155-5p was associated with reduced efflux of 5-FU.

OXA is also a commonly used chemotherapeutic drug that can be administered alone or in combination with fluorouracil. Yang et al. reported that CRC cells infused with resveratrol became more sensitive to OXA, which might have been related to the regulation of miR-34c [125]. Co-treatment with OXA and resveratrol showed enhanced efficacy of inhibition on tumor growth in mice. Resveratrol reduced OXA-induced side effects by inhibiting neurotoxicity and alleviating damage to the gastrointestinal mucosa [126]. Their combined treatment improved gastrointestinal contractility and diminished gastrointestinal dysfunction. Moreover, the resistance in colonic HCT116 cell lines was reduced by curcumin–OXA combination treatment through downregulation of the TGF-β/p-Smad2/3 pathway [127]. In vivo studies have also shown that mice dosed with the combination exhibited a more remarkable decrease in tumor weight and volume compared to those treated with curcumin or OXA alone. Alantolactone, a natural sesquiterpene lactone, enhanced OXA-induced cellular apoptosis in colonic cancer cells by increasing ROC generation and JNK/p38/MAPK pathway activation [128].

Irinotecan, a semisynthetic analog of camptothecin, has been shown to be beneficial against advanced and metastatic CRC. The EGCG–irinotecan combination caused more DNA damage, apoptosis induction, and suppression of cell migration in CRC cells than either agent alone [129]. Curcumin reversed the resistance of colonic cancer cells to irinotecan by inducing CSC apoptosis, as evidenced by the decreased expression of CSC-related biomarkers and colonosphere formation [130]. Curcumin also potentiated sensitivity to irinotecan in resistant colonic cancer cells through EMT regulation [131]. Moreover, the number of tumors decrease significantly in AOM/DSS-induced CRC model mice following co-treatment with GTE and irinotecan [132]. Additionally, GTE mitigated the side effects induced by irinotecan through the alleviation of neutropenia, leucopenia, and diarrhea in mice.

## 6. Discussion

The morbidity of CRC keeps rising along with changes in living environments and dietary habits, placing a heavy burden on medical resources and posing a great threat to public health. The application of polyphenols has attracted attention due to their low toxicity. Polyphenols act as chemopreventive agents to modulate multiple pathways in the suppression of cancer cell growth and metastasis. Among the different polyphenol types, some are relatively more studied in CRC, such as curcumin, resveratrol, EGCG, quercetin, apigenin, luteolin, anthocyanins, gallic acid, and capsaicin. The application of these polyphenols as adjuvant therapies would be a promising option for CRC prevention and treatment.

Accumulating evidence indicates a close association between natural polyphenols and the gut microbiota. Polyphenols and their metabolites can improve the intestinal microenvironment, thus intervening in CRC progression. Some gut-friendly bacteria are promoted while other undesirable bacteria are inhibited, which maintains the relative homeostasis of the intestinal microenvironment. Interestingly, new insights on the relationship between polyphenols and CSC have been shed in recent years. The development of CSCs may contribute to tumor formation, progression, and metastasis. Polyphenols regulate CRC-associated signaling pathways to exert anti-CRC effects.

Use of certain polyphenols alone only yields limited effects, but combination therapy would be more effective and safer. Polyphenols often have poor bioavailability and instability, which makes it difficult to maintain therapeutic concentrations within tumor cells and limits their wide application. Conventional chemotherapeutic drugs show varying degrees of toxicity to normal tissue, resulting in decreased sensitivity toward tumor cells. Co-treatment with polyphenols and other phytochemicals or chemotherapeutic drugs is beneficial to patients in that it could reduce the risk of adverse effects, lower the required dosage, and alleviate drug resistance. Importantly, combining different drugs requires properly adjusting doses to maximize their effectiveness without affecting their original function.

Efforts should be made in the following aspects. Studies of pharmacodynamics and network pharmacology should be conducted to identify new natural substances that are effective for CRC treatment. Despite achievements in elucidating the anti-cancer mechanisms of some polyphenols, they share different properties and characteristics that contribute to varied inhibitive effects. We should focus more on the compatibility of different natural polyphenols rather than on their individual effects, endeavoring to apply these agents more effectively and safely for the treatment of CRC and increasing their absorption in the human body. New drug-delivery regimens are also necessary, such as using nanoparticle formulations of targeted drugs, which could facilitate their uptake in tissues and improve their effectiveness against CRC cells. We recommend the combination of natural polyphenols with chemotherapy, which could not just enhance the “killability” of the agents against cancer cells, but also reduce the side effects of chemotherapy. Moreover, most of the relevant studies have been conducted in vitro or using animal models. Well-designed multi-centric, prospective, and randomized clinical trials are needed to further assess the efficacy of natural polyphenols in clinical settings. With the application in digital medicine, metagenomics sequencing and gene editing will become effective methods to identify more natural polyphenols for CRC-targeted therapy. Machine learning will help clinicians and researchers assess their long-term efficacy against CRC. Although polyphenols might only serve as adjuvant therapies against CRC, rather than the first-line agents, they have carved a new path for future CRC treatment and have broad prospects for development.

## 7. Conclusions

CRC remains one of the leading malignancies that poses a great threat to global public health and a heavy burden on society. Effective therapeutic agents are urgently needed to turn the table. Our study has found that an increasing number of natural polyphenols have been applied for CRC treatment and present remarkable anti-cancer activities. These agents can not only inhibit the growth and promote the death of CRC cells via regulation of multiple molecules and signaling pathways, but they exert anti-cancer effects through regulation of the gut microbiota and CSCs. It is worth noting that the combination of multiple polyphenols yields greater anti-cancer effects, and their combination with other plant-chemical substances of chemotherapeutic agents could be more beneficial to patients. However, limitations such as poor biological activity and stability should not be ignored. Therefore, more effective pharmaceutic preparations and delivery regimens are needed to extend their duration in the body. On the other hand, interdisciplinary cooperation using precision medicine and machine learning developed in the digital era can be applied to enable accurate identification of therapeutic targets and their long-term efficacy, thus promoting the development of new anti-CRC drugs that will benefit the whole of mankind.

## Figures and Tables

**Table 1:** The anti-CRC effects of different types of polyphenols.

Polyphenols	Type	Common Origin	Experimental model	Effect	Molecular Target	Reference
Curcumin	Other polyphenols	Plant *Curcuma longa*	SW480 cell line	Suppressed cell proliferation	Inhibition of Wnt/β-catenin pathway and downregulation of miRNA-130a	[34]
			SW480 and LoVo cell line	Inhibited cell adhesion and proliferation ability	Activation of AMPK and inhibition of NF-κB, uPA, and MMP9	[35]
			Rats	Slowed growth and altered distribution of liver metastases	N/A	[36]
Resveratrol	Other polyphenols	Mulberries, peanuts, and grapes	HCT116 and SW620 cell line	Induced cell apoptosis and decreased cell viability	Promotion of ROS generation and activation of mitochondrial apoptotic pathway	[37]
			LoVo cell line	Inhibited cell proliferation and induced cell apoptosis	Upregulation of BMP9 by MAPK pathway	[38]
			LoVo cell line	Suppressed cell invasion and metastasis	Inhibition of Wnt/β-catenin signaling and its downstream gene MALAT1	[39]
			Lung metastasis model of CRC in mice	Reduced formation of lung metastases, decreased tumor vascularization and tumor cell density	Reversal of EMT by AKT/GSK-3β/Snail pathway	[40]
EGCG	Flavonoids (flavanols)	Green tea	SW837 xenografts in nude mice	Suppressed cell growth and tumor angiogenesis	Inhibition of HIF-1alpha and VEGF	[41]
			SW480, HCT116 and Caco2 cell line BALB/c nude mice	Triggered cell apoptosis and decreased tumor weight	Downregulation of PI3K/AKT pathway	[42]
			DMH-induced CRC model of rats	Reduced tumor formation rate, tumor number, tumor volume, precancerous lesion and ascites formation	N/A	[43]
Quercetin	Flavonoids (flavonols)	Onions, asparagus, and berries	CT26 and MC38 cell line	Induced apoptosis and reduced cell migration	Activation of MAPK pathway and suppression of MMP-2 and MMP-9	[44]
			HT29 and HCT15 cell line	Induced apoptosis and inhibited cell survival	Increased ROS generation by promoting COX-2	[45]
			SW480 cell line	Reduced cell viability and induced apoptosis	Downregulation of Wnt/β-catenin pathway and its target genes (cyclin D1 and survivin)	[46]
			AOM/DSS-induced CRC model in mice	Reduced tumor size, attenuated inflammation, and downregulated oxidative stress markers	N/A	[47]
Apigenin	Flavonoids (flavones)	Celery, parsley, chamomile	SW480, DLD-1 and LS174T cell line	Inhibited cell proliferation and migration	Upregulation of transgelin expression and downregulation of MMP-9 expression by mediating Akt pathway	[48]
			DLD1 and SW480 cell line	Inhibited migration and invasion	Downregulation of NEDD9 expression to suppress Akt pathway	[49]
			HCT-116, SW480, HT-29 and LoVo cell line APC^Min/+^ mice	Enhanced cell growth arrest and apoptosis, reduced polyp numbers	Promotion of pro-apoptotic protein (NAG-1 and p53) and cell cycle inhibitor (p21) by mediating PKCδ pathway	[50]
			HCT-8 cell line	Inhibited tumor angiogenesis	Repression of HIF-1 and VEGF	[51]
Luteolin	Flavonoids (flavones)	Plant families of Pteridophyta, Bryophyta, Magnoliophyta, and Pinophyta	COLO205 and HCT116 cell line	Induced apoptosis	Sensitization of TNF-alpha and suppression of NF-κB and its targeted genes	[52]
			HCT-15 cell line	Promoted cell cycle arrest and apoptosis	Inhibition of glycogen synthase kinase-3β and cyclin D1 through Wnt/β-catenin pathway	[53]
			AOM-induced CRC mice	Decreased levels of metastatic tumor markers	Inhibition of MMP-2 and MMP-9, and promotion of TIMP-2 expression	[54]
			HT-29 and SNU-407 cell line	Promoted apoptosis induction	Promotion of DNA demethylation by Nrf2 pathway	[55]
Anthocyanins	Flavonoids	Vegetables, fruits, and wine	HCT-116 cell line	Inhibited cell viability and promoted cell apoptosis	Upregulation of p38-MAPK and downregulation of Akt	[56]
			HCT-116 cell line	Attenuated cell migration	Modulation of TJ and downregulation of MMP expression through upregulation of p38-MAPK and downregulation of Akt	[57]
			Caco-2 cell line	Inhibited cell proliferation	Upregulation of p21Waf/Cif1, activation of caspase-3, and promotion of ROS generation	[58]
			AOM-induced CRC mice	Inhibited the incidence of inflammation and promotion of colon tumors	Repression of proinflammatory mediators and oncogenic pathways, including PI3K, Akt, ERK, and NF-κB	[59]
Gallic acid	Phenolic acids	Certain red fruits, black radish, and onion	HCT-15 cell line	Caused cell cycle arrest, cell apoptosis, and reduced colony formation	Activation of ROS-mitochondrial pathway	[60]
			CRL1790, SW480 and SW620 cell line CRC model in mice	Inhibited cell proliferation, increased DNA damage, decreased Ki67 expression and tumor volume	Interaction with DNA G4s	[61]
			DMH-induced CRC rats	Suppressed colon carcinogenesis	Upregulation of lipid peroxidation and antioxidant expression	[62]
			DMH-induced CRC rats	Reduced tumor incidence	Inhibition of phase I enzymes and promotion of phase II enzyme activities	[63]
Capsaicin	Polyphenolic amides	Chili peppers	Colo320DM and LoVo cell line	Decreased cell viability and induced cell apoptosis	Enhanced accumulation of ROS and caspase-3 activation	[64]
			HT-29 cell line	Increased cell apoptosis	Activation of AMPK pathway	[65]
			SW480, LoVo, and HCT-116 cell line	Reduced cell proliferation	Suppression of β-catenin expression and its binding to TCF	[66]
			DMH-induced CRC rats	Decreased the number and multiplicity of ACF	N/A	[67]
coffee polyphenols (chlorogenic acid)	Phenolic acids	coffee	HT-29 cell line and SW480 cell line	Decreased activity of CRC cells and inhibited migration and invasion	Inhibition of CTNNB1,CDH1 CCND1, activation of CRC cells, and regulation of Wnt/β-catenin pathway	[68]
ferulic acid	Phenolic acid	Plant	HCT-116 and Caco2 cells	Anti-angiogenesis and promoted CRC cell apoptosis	Downregulation of cyclin D1, IGF II, and VEGF, regulation of BAX/BcI-2 genes	[69]
EPE	Phenolic acid, Flavonoids	evening primrose seeds	CRC cell lines and AOM-DSS-induced colitis-associated colon cancer in mice	Inhibited invasion and metastasis of cancer cells	Downregulation of TYMS	[70]
kaempferol	Flavonoids	Kaempferol L	Apc^Min/+^ mice	Reduced CRC tumor load and restored intestinal mucosal barrier	Downregulation of Ki67 and LGR5 and regulation of bile acid secretion	[71]

uPA: urokinase-type plasminogen activator; MMP9: matrix metalloproteinase-9; ROS: reactive oxygen species; BMP9: bone morphogenetic protein 9; EMT: epithelial-mesenchymal transition; HIF: hypoxia-inducible factor; VEGF: vascular endothelial growth factor; DMH: dimethylhydrazine; AOM: azoxymethane; COX-2: cyclooxygenase-2; DSS: dextran sodium sulfate; NAG-1: non-steroidal anti-inflammatory drug (NSAID)-activated gene-1; Min, multiple intestinal neoplasia; TNF: tumor necrosis factor; TIMP: tissue inhibitor of matrix metalloproteinase; TJ: tight junction; G4s: G-quadruplexes; TCF: T-cell factor; ACF: aberrant crypt foci; N/A: not applicable.

## Data Availability

No new data were created or analyzed in this study. Data sharing is not applicable to this article.

## References

[1] Sung H., Ferlay J., Siegel R.L., Laversanne M., Soerjomataram I., Jemal A., Bray F. (2021). Global Cancer Statistics 2020: GLOBOCAN Estimates of Incidence and Mortality Worldwide for 36 Cancers in 185 Countries. CA Cancer J. Clin..

[2] Li N., Lu B., Luo C., Cai J., Lu M., Zhang Y., Chen H., Dai M. (2021). Incidence, mortality, survival, risk factor and screening of colorectal cancer: A comparison among China, Europe, and northern America. Cancer Lett..

[3] Anwar S., Shamsi A., Shahbaaz M., Queen A., Khan P., Hasan G.M., Islam A., Alajmi M.F., Hussain A., Ahmad F. (2020). Rosmarinic Acid Exhibits Anticancer Effects via MARK4 Inhibition. Sci. Rep..

[4] Zhou Y., Zheng J., Li Y., Xu D.P., Li S., Chen Y.M., Li H.B. (2016). Natural Polyphenols for Prevention and Treatment of Cancer. Nutrients.

[5] Tjalsma H., Boleij A., Marchesi J.R., Dutilh B.E. (2012). A bacterial driver-passenger model for colorectal cancer: Beyond the usual suspects. Nat. Rev. Microbiol..

[6] Rubinstein M.R., Wang X., Liu W., Hao Y., Cai G., Han Y.W. (2013). Fusobacterium nucleatum promotes colorectal carcinogenesis by modulating E-cadherin/β-catenin signaling via its FadA adhesin. Cell Host Microbe.

[7] Huycke M.M., Abrams V., Moore D.R. (2002). Enterococcus faecalis produces extracellular superoxide and hydrogen peroxide that damages colonic epithelial cell DNA. Carcinogenesis.

[8] Dalal N., Jalandra R., Bayal N., Yadav A.K., Harshulika, Sharma M., Makharia G.K., Kumar P., Singh R., Solanki P.R. (2021). Gut microbiota-derived metabolites in CRC progression and causation. J. Cancer Res. Clin. Oncol..

[9] Jacouton E., Chain F., Sokol H., Langella P., Bermúdez-Humarán L.G. (2017). Probiotic Strain Lactobacillus casei BL23 Prevents Colitis-Associated Colorectal Cancer. Front. Immunol..

[10] Wang J., Tang L., Zhou H., Zhou J., Glenn T.C., Shen C.L., Wang J.S. (2018). Long-term treatment with green tea polyphenols modifies the gut microbiome of female sprague-dawley rats. J. Nutr. Biochem..

[11] Li F., Han Y., Cai X., Gu M., Sun J., Qi C., Goulette T., Song M., Li Z., Xiao H. (2020). Dietary resveratrol attenuated colitis and modulated gut microbiota in dextran sulfate sodium-treated mice. Food Funct..

[12] Lacombe A., Li R.W., Klimis-Zacas D., Kristo A.S., Tadepalli S., Krauss E., Young R., Wu V.C. (2013). Lowbush wild blueberries have the potential to modify gut microbiota and xenobiotic metabolism in the rat colon. PLoS ONE.

[13] Wan M.L.Y., Co V.A., El-Nezami H. (2021). Dietary polyphenol impact on gut health and microbiota. Crit. Rev. Food Sci. Nutr..

[14] Sorrenti V., Ali S., Mancin L., Davinelli S., Paoli A., Scapagnini G. (2020). Cocoa Polyphenols and Gut Microbiota Interplay: Bioavailability, Prebiotic Effect, and Impact on Human Health. Nutrients.

[15] Wang X., Ye T., Chen W.J., Lv Y., Hao Z., Chen J., Zhao J.Y., Wang H.P., Cai Y.K. (2017). Structural shift of gut microbiota during chemo-preventive effects of epigallocatechin gallate on colorectal carcinogenesis in mice. World J. Gastroenterol..

[16] Chen L., Jiang B., Zhong C., Guo J., Zhang L., Mu T., Zhang Q., Bi X. (2018). Chemoprevention of colorectal cancer by black raspberry anthocyanins involved the modulation of gut microbiota and SFRP2 demethylation. Carcinogenesis.

[17] McFadden R.M., Larmonier C.B., Shehab K.W., Midura-Kiela M., Ramalingam R., Harrison C.A., Besselsen D.G., Chase J.H., Caporaso J.G., Jobin C. (2015). The Role of Curcumin in Modulating Colonic Microbiota During Colitis and Colon Cancer Prevention. Inflamm. Bowel Dis..

[18] Molan A.L., Liu Z., Tiwari R. (2010). The ability of green tea to positively modulate key markers of gastrointestinal function in rats. Phytother. Res. PTR.

[19] Han Y., Haraguchi T., Iwanaga S., Tomotake H., Okazaki Y., Mineo S., Moriyama A., Inoue J., Kato N. (2009). Consumption of some polyphenols reduces fecal deoxycholic acid and lithocholic acid, the secondary bile acids of risk factors of colon cancer. J. Agric. Food Chem..

[20] Bernstein H., Bernstein C., Payne C.M., Dvorakova K., Garewal H. (2005). Bile acids as carcinogens in human gastrointestinal cancers. Mutat. Res..

[21] Rodríguez-Morató J., Matthan N.R., Liu J., de la Torre R., Chen C.O. (2018). Cranberries attenuate animal-based diet-induced changes in microbiota composition and functionality: A randomized crossover controlled feeding trial. J. Nutr. Biochem..

[22] Zhang Z., Peng X., Zhang N., Liu L., Wang Y., Ou S. (2014). Cytotoxicity comparison of quercetin and its metabolites from in vitro fermentation of several gut bacteria. Food Funct..

[23] Aires V., Limagne E., Cotte A.K., Latruffe N., Ghiringhelli F., Delmas D. (2013). Resveratrol metabolites inhibit human metastatic colon cancer cells progression and synergize with chemotherapeutic drugs to induce cell death. Mol. Nutr. Food Res..

[24] Núñez-Sánchez M., Karmokar A., González-Sarrías A., García-Villalba R., Tomás-Barberán F.A., García-Conesa M.T., Brown K., Espín J.C. (2016). In vivo relevant mixed urolithins and ellagic acid inhibit phenotypic and molecular colon cancer stem cell features: A new potentiality for ellagitannin metabolites against cancer. Food Chem. Toxicol..

[25] Du L., Cheng Q., Zheng H., Liu J., Liu L., Chen Q. (2022). Targeting stemness of cancer stem cells to fight colorectal cancers. Semin. Cancer Biol..

[26] Ganesan K., Jayachandran M., Xu B. (2020). Diet-Derived Phytochemicals Targeting Colon Cancer Stem Cells and Microbiota in Colorectal Cancer. Int. J. Mol. Sci..

[27] Lin L., Liu Y., Li H., Li P.K., Fuchs J., Shibata H., Iwabuchi Y., Lin J. (2011). Targeting colon cancer stem cells using a new curcumin analogue, GO-Y030. British journal of cancer.

[28] Kanwar S.S., Yu Y., Nautiyal J., Patel B.B., Padhye S., Sarkar F.H., Majumdar A.P. (2011). Difluorinated-curcumin (CDF): A novel curcumin analog is a potent inhibitor of colon cancer stem-like cells. Pharm. Res..

[29] Roy S., Yu Y., Padhye S.B., Sarkar F.H., Majumdar A.P. (2013). Difluorinated-curcumin (CDF) restores PTEN expression in colon cancer cells by down-regulating miR-21. PLoS ONE.

[30] Toden S., Tran H.M., Tovar-Camargo O.A., Okugawa Y., Goel A. (2016). Epigallocatechin-3-gallate targets cancer stem-like cells and enhances 5-fluorouracil chemosensitivity in colorectal cancer. Oncotarget.

[31] Chen Y., Wang X.Q., Zhang Q., Zhu J.Y., Li Y., Xie C.F., Li X.T., Wu J.S., Geng S.S., Zhong C.Y. (2017). (-)-Epigallocatechin-3-Gallate Inhibits Colorectal Cancer Stem Cells by Suppressing Wnt/β-Catenin Pathway. Nutrients.

[32] Reddivari L., Charepalli V., Radhakrishnan S., Vadde R., Elias R.J., Lambert J.D., Vanamala J.K. (2016). Grape compounds suppress colon cancer stem cells in vitro and in a rodent model of colon carcinogenesis. BMC Complement. Altern. Med..

[33] Pouyafar A., Rezabakhsh A., Rahbarghazi R., Heydarabad M.Z., Shokrollahi E., Sokullu E., Khaksar M., Nourazarian A., Avci Ç.B. (2019). Treatment of cancer stem cells from human colon adenocarcinoma cell line HT-29 with resveratrol and sulindac induced mesenchymal-endothelial transition rate. Cell Tissue Res..

[34] Dou H., Shen R., Tao J., Huang L., Shi H., Chen H., Wang Y., Wang T. (2017). Curcumin Suppresses the Colon Cancer Proliferation by Inhibiting Wnt/β-Catenin Pathways via miR-130a. Front. Pharmacol..

[35] Tong W., Wang Q., Sun D., Suo J. (2016). Curcumin suppresses colon cancer cell invasion via AMPK-induced inhibition of NF-κB, uPA activator and MMP9. Oncol. Lett..

[36] Herrero de la Parte B., Rodeño-Casado M., Iturrizaga Correcher S., Mar Medina C., García-Alonso I. (2021). Curcumin Reduces Colorectal Cancer Cell Proliferation and Migration and Slows In Vivo Growth of Liver Metastases in Rats. Biomedicines.

[37] Fu Y., Ye Y., Zhu G., Xu Y., Sun J., Wu H., Feng F., Wen Z., Jiang S., Li Y. (2021). Resveratrol induces human colorectal cancer cell apoptosis by activating the mitochondrial pathway via increasing reactive oxygen species. Mol. Med. Rep..

[38] Yuan S.X., Wang D.X., Wu Q.X., Ren C.M., Li Y., Chen Q.Z., Zeng Y.H., Shao Y., Yang J.Q., Bai Y. (2016). BMP9/p38 MAPK is essential for the antiproliferative effect of resveratrol on human colon cancer. Oncol. Rep..

[39] Ji Q., Liu X., Fu X., Zhang L., Sui H., Zhou L., Sun J., Cai J., Qin J., Ren J. (2013). Resveratrol inhibits invasion and metastasis of colorectal cancer cells via MALAT1 mediated Wnt/β-catenin signal pathway. PLoS ONE.

[40] Yuan L., Zhou M., Huang D., Wasan H.S., Zhang K., Sun L., Huang H., Ma S., Shen M., Ruan S. (2019). Resveratrol inhibits the invasion and metastasis of colon cancer through reversal of epithelial- mesenchymal transition via the AKT/GSK-3β/Snail signaling pathway. Mol. Med. Rep..

[41] Md Nesran Z.N., Shafie N.H., Ishak A.H., Mohd Esa N., Ismail A., Md Tohid S.F. (2019). Induction of Endoplasmic Reticulum Stress Pathway by Green Tea Epigallocatechin-3-Gallate (EGCG) in Colorectal Cancer Cells: Activation of PERK/p-eIF2α/ATF4 and IRE1α. BioMed Res. Int..

[42] Shimizu M., Shirakami Y., Sakai H., Yasuda Y., Kubota M., Adachi S., Tsurumi H., Hara Y., Moriwaki H. (2010). (-)-Epigallocatechin gallate inhibits growth and activation of the VEGF/VEGFR axis in human colorectal cancer cells. Chemico-Biol. Interact..

[43] Wang Y., Jin H.Y., Fang M.Z., Wang X.F., Chen H., Huang S.L., Kong D.S., Li M., Zhang X., Sun Y. (2020). Epigallocatechin gallate inhibits dimethylhydrazine-induced colorectal cancer in rats. World J. Gastroenterol..

[44] Kee J.Y., Han Y.H., Kim D.S., Mun J.G., Park J., Jeong M.Y., Um J.Y., Hong S.H. (2016). Inhibitory effect of quercetin on colorectal lung metastasis through inducing apoptosis, and suppression of metastatic ability. Phytomedicine.

[45] Raja S.B., Rajendiran V., Kasinathan N.K., P A., Venkatabalasubramanian S., Murali M.R., Devaraj H., Devaraj S.N. (2017). Differential cytotoxic activity of Quercetin on colonic cancer cells depends on ROS generation through COX-2 expression. Food Chem. Toxicol..

[46] Shan B.E., Wang M.X., Li R.Q. (2009). Quercetin inhibit human SW480 colon cancer growth in association with inhibition of cyclin D1 and survivin expression through Wnt/beta-catenin signaling pathway. Cancer Investig..

[47] Lin R., Piao M., Song Y., Liu C. (2020). Quercetin Suppresses AOM/DSS-Induced Colon Carcinogenesis through Its Anti-Inflammation Effects in Mice. J. Immunol. Res..

[48] Chunhua L., Donglan L., Xiuqiong F., Lihua Z., Qin F., Yawei L., Liang Z., Ge W., Linlin J., Ping Z. (2013). Apigenin up-regulates transgelin and inhibits invasion and migration of colorectal cancer through decreased phosphorylation of AKT. J. Nutr. Biochem..

[49] Dai J., Van Wie P.G., Fai L.Y., Kim D., Wang L., Poyil P., Luo J., Zhang Z. (2016). Downregulation of NEDD9 by apigenin suppresses migration, invasion, and metastasis of colorectal cancer cells. Toxicol. Appl. Pharmacol..

[50] Zhong Y., Krisanapun C., Lee S.H., Nualsanit T., Sams C., Peungvicha P., Baek S.J. (2010). Molecular targets of apigenin in colorectal cancer cells: Involvement of p21, NAG-1 and p53. Eur. J. Cancer.

[51] Fang J., Zhou Q., Liu L.Z., Xia C., Hu X., Shi X., Jiang B.H. (2007). Apigenin inhibits tumor angiogenesis through decreasing HIF-1alpha and VEGF expression. Carcinogenesis.

[52] Shi R.X., Ong C.N., Shen H.M. (2004). Luteolin sensitizes tumor necrosis factor-alpha-induced apoptosis in human tumor cells. Oncogene.

[53] Pandurangan A.K., Dharmalingam P., Sadagopan S.K., Ramar M., Munusamy A., Ganapasam S. (2013). Luteolin induces growth arrest in colon cancer cells through involvement of Wnt/β-catenin/GSK-3β signaling. J. Environ. Pathol. Toxicol. Oncol..

[54] Pandurangan A.K., Dharmalingam P., Sadagopan S.K., Ganapasam S. (2014). Luteolin inhibits matrix metalloproteinase 9 and 2 in azoxymethane-induced colon carcinogenesis. Hum. Exp. Toxicol..

[55] Kang K.A., Piao M.J., Hyun Y.J., Zhen A.X., Cho S.J., Ahn M.J., Yi J.M., Hyun J.W. (2019). Luteolin promotes apoptotic cell death via upregulation of Nrf2 expression by DNA demethylase and the interaction of Nrf2 with p53 in human colon cancer cells. Exp. Mol. Med..

[56] Shin D.Y., Lee W.S., Lu J.N., Kang M.H., Ryu C.H., Kim G.Y., Kang H.S., Shin S.C., Choi Y.H. (2009). Induction of apoptosis in human colon cancer HCT-116 cells by anthocyanins through suppression of Akt and activation of p38-MAPK. Int. J. Oncol..

[57] Shin D.Y., Lu J.N., Kim G.Y., Jung J.M., Kang H.S., Lee W.S., Choi Y.H. (2011). Anti-invasive activities of anthocyanins through modulation of tight junctions and suppression of matrix metalloproteinase activities in HCT-116 human colon carcinoma cells. Oncol. Rep..

[58] Anwar S., Fratantonio D., Ferrari D., Saija A., Cimino F., Speciale A. (2016). Berry anthocyanins reduce proliferation of human colorectal carcinoma cells by inducing caspase-3 activation and p21 upregulation. Mol. Med. Rep..

[59] Shi N., Clinton S.K., Liu Z., Wang Y., Riedl K.M., Schwartz S.J., Zhang X., Pan Z., Chen T. (2015). Strawberry phytochemicals inhibit azoxymethane/dextran sodium sulfate-induced colorectal carcinogenesis in Crj: CD-1 mice. Nutrients.

[60] Subramanian A.P., Jaganathan S.K., Mandal M., Supriyanto E., Muhamad I.I. (2016). Gallic acid induced apoptotic events in HCT-15 colon cancer cells. World J. Gastroenterol..

[61] Sanchez-Martin V., Plaza-Calonge M.D.C., Soriano-Lerma A., Ortiz-Gonzalez M., Linde-Rodriguez A., Perez-Carrasco V., Ramirez-Macias I., Cuadros M., Gutierrez-Fernandez J., Murciano-Calles J. (2022). Gallic Acid: A Natural Phenolic Compound Exerting Antitumoral Activities in Colorectal Cancer via Interaction with G-Quadruplexes. Cancers.

[62] Giftson J.S., Jayanthi S., Nalini N. (2010). Chemopreventive efficacy of gallic acid, an antioxidant and anticarcinogenic polyphenol, against 1,2-dimethyl hydrazine induced rat colon carcinogenesis. Investig. New Drugs.

[63] Giftson Senapathy J., Jayanthi S., Viswanathan P., Umadevi P., Nalini N. (2011). Effect of gallic acid on xenobiotic metabolizing enzymes in 1,2-dimethyl hydrazine induced colon carcinogenesis in Wistar rats—A chemopreventive approach. Food Chem. Toxicol..

[64] Yang K.M., Pyo J.O., Kim G.Y., Yu R., Han I.S., Ju S.A., Kim W.H., Kim B.S. (2009). Capsaicin induces apoptosis by generating reactive oxygen species and disrupting mitochondrial transmembrane potential in human colon cancer cell lines. Cell. Mol. Biol. Lett..

[65] Kim Y.M., Hwang J.T., Kwak D.W., Lee Y.K., Park O.J. (2007). Involvement of AMPK signaling cascade in capsaicin-induced apoptosis of HT-29 colon cancer cells. Ann. N. Y. Acad. Sci..

[66] Lee S.H., Richardson R.L., Dashwood R.H., Baek S.J. (2012). Capsaicin represses transcriptional activity of β-catenin in human colorectal cancer cells. J. Nutr. Biochem..

[67] Caetano B.F.R., Tablas M.B., Pereira N.E.F., de Moura N.A., Carvalho R.F., Rodrigues M.A.M., Barbisan L.F. (2018). Capsaicin reduces genotoxicity, colonic cell proliferation and preneoplastic lesions induced by 1,2-dimethylhydrazine in rats. Toxicol. Appl. Pharmacol..

[68] Villota H., Santa-González G.A., Uribe D., Henao I.C., Arroyave-Ospina J.C., Barrera-Causil C.J., Pedroza-Díaz J. (2022). Modulatory Effect of Chlorogenic Acid and Coffee Extracts on Wnt/β-Catenin Pathway in Colorectal Cancer Cells. Nutrients.

[69] El-Gogary R.I., Nasr M., Rahsed L.A., Hamzawy M.A. (2022). Ferulic acid nanocapsules as a promising treatment modality for colorectal cancer: Preparation and in vitro/in vivo appraisal. Life Sci..

[70] Ciszewski W.M., Włodarczyk J., Chmielewska-Kassassir M., Fichna J., Wozniak L.A., Sobierajska K. (2022). Evening primrose seed extract rich in polyphenols modulates the invasiveness of colon cancer cells by regulating the TYMS expression. Food Funct..

[71] Li X., Khan I., Huang G., Lu Y., Wang L., Liu Y., Lu L., Hsiao W.L.W., Liu Z. (2022). Kaempferol acts on bile acid signaling and gut microbiota to attenuate the tumor burden in ApcMin/+ mice. Eur. J. Pharmacol..

[72] Bahrami A., Amerizadeh F., ShahidSales S., Khazaei M., Ghayour-Mobarhan M., Sadeghnia H.R., Maftouh M., Hassanian S.M., Avan A. (2017). Therapeutic Potential of Targeting Wnt/β-Catenin Pathway in Treatment of Colorectal Cancer: Rational and Progress. J. Cell. Biochem..

[73] Ruiz de Porras V., Bystrup S., Martínez-Cardús A., Pluvinet R., Sumoy L., Howells L., James M.I., Iwuji C., Manzano J.L., Layos L. (2016). Curcumin mediates oxaliplatin-acquired resistance reversion in colorectal cancer cell lines through modulation of CXC-Chemokine/NF-κB signalling pathway. Sci. Rep..

[74] Howells L.M., Iwuji C.O.O., Irving G.R.B., Barber S., Walter H., Sidat Z., Griffin-Teall N., Singh R., Foreman N., Patel S.R. (2019). Curcumin Combined with FOLFOX Chemotherapy Is Safe and Tolerable in Patients with Metastatic Colorectal Cancer in a Randomized Phase IIa Trial. J. Nutr..

[75] James M.I., Iwuji C., Irving G., Karmokar A., Higgins J.A., Griffin-Teal N., Thomas A., Greaves P., Cai H., Patel S.R. (2015). Curcumin inhibits cancer stem cell phenotypes in ex vivo models of colorectal liver metastases, and is clinically safe and tolerable in combination with FOLFOX chemotherapy. Cancer Lett..

[76] Vernousfaderani E.K., Akhtari N., Rezaei S., Rezaee Y., Shiranirad S., Mashhadi M., Hashemi A., Khankandi H.P., Behzad S. (2021). Resveratrol and Colorectal Cancer: A Molecular Approach to Clinical Researches. Curr. Top. Med. Chem..

[77] Patel K.R., Brown V.A., Jones D.J., Britton R.G., Hemingway D., Miller A.S., West K.P., Booth T.D., Perloff M., Crowell J.A. (2010). Clinical pharmacology of resveratrol and its metabolites in colorectal cancer patients. Cancer Res..

[78] Howells L.M., Berry D.P., Elliott P.J., Jacobson E.W., Hoffmann E., Hegarty B., Brown K., Steward W.P., Gescher A.J. (2011). Phase I randomized, double-blind pilot study of micronized resveratrol (SRT501) in patients with hepatic metastases--safety, pharmacokinetics, and pharmacodynamics. Cancer Prev. Res..

[79] Ding F., Yang S. (2021). Epigallocatechin-3-gallate inhibits proliferation and triggers apoptosis in colon cancer via the hedgehog/phosphoinositide 3-kinase pathways. Can. J. Physiol. Pharmacol..

[80] Jin H., Gong W., Zhang C., Wang S. (2013). Epigallocatechin gallate inhibits the proliferation of colorectal cancer cells by regulating Notch signaling. OncoTargets Ther..

[81] Cerezo-Guisado M.I., Zur R., Lorenzo M.J., Risco A., Martín-Serrano M.A., Alvarez-Barrientos A., Cuenda A., Centeno F. (2015). Implication of Akt, ERK1/2 and alternative p38MAPK signalling pathways in human colon cancer cell apoptosis induced by green tea EGCG. Food Chem. Toxicol..

[82] Hwang J.T., Ha J., Park I.J., Lee S.K., Baik H.W., Kim Y.M., Park O.J. (2007). Apoptotic effect of EGCG in HT-29 colon cancer cells via AMPK signal pathway. Cancer Lett..

[83] Hu Y., McIntosh G.H., Le Leu R.K., Somashekar R., Meng X.Q., Gopalsamy G., Bambaca L., McKinnon R.A., Young G.P. (2016). Supplementation with Brazil nuts and green tea extract regulates targeted biomarkers related to colorectal cancer risk in humans. Br. J. Nutr..

[84] Sinicrope F.A., Viggiano T.R., Buttar N.S., Song L., Schroeder K.W., Kraichely R.E., Larson M.V., Sedlack R.E., Kisiel J.B., Gostout C.J. (2021). Randomized Phase II Trial of Polyphenon E versus Placebo in Patients at High Risk of Recurrent Colonic Neoplasia. Cancer Prev. Res..

[85] Seufferlein T., Ettrich T.J., Menzler S., Messmann H., Kleber G., Zipprich A., Frank-Gleich S., Algül H., Metter K., Odemar F. (2022). Green Tea Extract to Prevent Colorectal Adenomas, Results of a Randomized, Placebo-Controlled Clinical Trial. Am. J. Gastroenterol..

[86] Refolo M.G., D’Alessandro R., Malerba N., Laezza C., Bifulco M., Messa C., Caruso M.G., Notarnicola M., Tutino V. (2015). Anti Proliferative and Pro Apoptotic Effects of Flavonoid Quercetin Are Mediated by CB1 Receptor in Human Colon Cancer Cell Lines. J. Cell. Physiol..

[87] Zhang X.A., Zhang S., Yin Q., Zhang J. (2015). Quercetin induces human colon cancer cells apoptosis by inhibiting the nuclear factor-kappa B Pathway. Pharmacogn. Mag..

[88] Cruz-Correa M., Shoskes D.A., Sanchez P., Zhao R., Hylind L.M., Wexner S.D., Giardiello F.M. (2006). Combination treatment with curcumin and quercetin of adenomas in familial adenomatous polyposis. Clin. Gastroenterol. Hepatol..

[89] Bobe G., Albert P.S., Sansbury L.B., Lanza E., Schatzkin A., Colburn N.H., Cross A.J. (2010). Interleukin-6 as a potential indicator for prevention of high-risk adenoma recurrence by dietary flavonols in the polyp prevention trial. Cancer Prev. Res..

[90] Chang H., Lei L., Zhou Y., Ye F., Zhao G. (2018). Dietary Flavonoids and the Risk of Colorectal Cancer: An Updated Meta-Analysis of Epidemiological Studies. Nutrients.

[91] Tong J., Shen Y., Zhang Z., Hu Y., Zhang X., Han L. (2019). Apigenin inhibits epithelial-mesenchymal transition of human colon cancer cells through NF-κB/Snail signaling pathway. Biosci. Rep..

[92] Chen X., Xu H., Yu X., Wang X., Zhu X., Xu X. (2019). Apigenin inhibits in vitro and in vivo tumorigenesis in cisplatin-resistant colon cancer cells by inducing autophagy, programmed cell death and targeting m-TOR/PI3K/Akt signalling pathway. J. B.U.ON..

[93] Lin C.M., Chen H.H., Lin C.A., Wu H.C., Sheu J.J., Chen H.J. (2017). Apigenin-induced lysosomal degradation of β-catenin in Wnt/β-catenin signaling. Sci. Rep..

[94] Hoensch H., Groh B., Edler L., Kirch W. (2008). Prospective cohort comparison of flavonoid treatment in patients with resected colorectal cancer to prevent recurrence. World J. Gastroenterol..

[95] Pandurangan A.K., Dharmalingam P., Ananda Sadagopan S.K., Ganapasam S. (2012). Effect of luteolin on the levels of glycoproteins during azoxymethane-induced colon carcinogenesis in mice. Asian Pac. J. Cancer Prev. APJCP.

[96] Pandurangan A.K., Ananda Sadagopan S.K., Dharmalingam P., Ganapasam S. (2014). Luteolin, a bioflavonoid inhibits Azoxymethane-induced colorectal cancer through activation of Nrf2 signaling. Toxicol. Mech. Methods.

[97] Khor T.O., Huang M.T., Prawan A., Liu Y., Hao X., Yu S., Cheung W.K., Chan J.Y., Reddy B.S., Yang C.S. (2008). Increased susceptibility of Nrf2 knockout mice to colitis-associated colorectal cancer. Cancer Prev. Res..

[98] Yoo H.S., Won S.B., Kwon Y.H. (2022). Luteolin Induces Apoptosis and Autophagy in HCT116 Colon Cancer Cells via p53-Dependent Pathway. Nutr. Cancer.

[99] Kang K.A., Piao M.J., Ryu Y.S., Hyun Y.J., Park J.E., Shilnikova K., Zhen A.X., Kang H.K., Koh Y.S., Jeong Y.J. (2017). Luteolin induces apoptotic cell death via antioxidant activity in human colon cancer cells. Int. J. Oncol..

[100] Kim H.Y., Jung S.K., Byun S., Son J.E., Oh M.H., Lee J., Kang M.J., Heo Y.S., Lee K.W., Lee H.J. (2013). Raf and PI3K are the molecular targets for the anti-metastatic effect of luteolin. Phytother. Res. PTR.

[101] Lim D.Y., Cho H.J., Kim J., Nho C.W., Lee K.W., Park J.H. (2012). Luteolin decreases IGF-II production and downregulates insulin-like growth factor-I receptor signaling in HT-29 human colon cancer cells. BMC Gastroenterol..

[102] Potočnjak I., Šimić L., Gobin I., Vukelić I., Domitrović R. (2020). Antitumor activity of luteolin in human colon cancer SW620 cells is mediated by the ERK/FOXO3a signaling pathway. Toxicol. In Vitro.

[103] Yao Y., Rao C., Zheng G., Wang S. (2019). Luteolin suppresses colorectal cancer cell metastasis via regulation of the miR-384/pleiotrophin axis. Oncol. Rep..

[104] Wang X., Yang D.Y., Yang L.Q., Zhao W.Z., Cai L.Y., Shi H.P. (2019). Anthocyanin Consumption and Risk of Colorectal Cancer: A Meta-Analysis of Observational Studies. J. Am. Coll. Nutr..

[105] Wei J., Yu W., Hao R., Fan J., Gao J. (2020). Anthocyanins from Aronia melanocarpa Induce Apoptosis in Caco-2 Cells through Wnt/β-Catenin Signaling Pathway. Chem. Biodivers..

[106] Charepalli V., Reddivari L., Radhakrishnan S., Vadde R., Agarwal R., Vanamala J.K. (2015). Anthocyanin-containing purple-fleshed potatoes suppress colon tumorigenesis via elimination of colon cancer stem cells. J. Nutr. Biochem..

[107] Wang L.S., Kuo C.T., Cho S.J., Seguin C., Siddiqui J., Stoner K., Weng Y.I., Huang T.H., Tichelaar J., Yearsley M. (2013). Black raspberry-derived anthocyanins demethylate tumor suppressor genes through the inhibition of DNMT1 and DNMT3B in colon cancer cells. Nutr. Cancer.

[108] Asadi K., Ferguson L.R., Philpott M., Karunasinghe N. (2017). Cancer-preventive Properties of an Anthocyanin-enriched Sweet Potato in the APC(MIN) Mouse Model. J. Cancer Prev..

[109] Koyuncu İ., Gönel A., Temiz E., Karaoğul E., Uyar Z. (2021). Pistachio Green Hull Extract Induces Apoptosis through Multiple Signaling Pathways by Causing Oxidative Stress on Colon Cancer Cells. Anti-Cancer Agents Med. Chem..

[110] Kosiol N., Juranek S., Brossart P., Heine A., Paeschke K. (2021). G-quadruplexes: A promising target for cancer therapy. Mol. Cancer.

[111] Lu H.F., Chen Y.L., Yang J.S., Yang Y.Y., Liu J.Y., Hsu S.C., Lai K.C., Chung J.G. (2010). Antitumor activity of capsaicin on human colon cancer cells in vitro and colo 205 tumor xenografts in vivo. J. Agric. Food Chem..

[112] Lee S.H., Krisanapun C., Baek S.J. (2010). NSAID-activated gene-1 as a molecular target for capsaicin-induced apoptosis through a novel molecular mechanism involving GSK3beta, C/EBPbeta and ATF3. Carcinogenesis.

[113] Yang J., Li T.Z., Xu G.H., Luo B.B., Chen Y.X., Zhang T. (2013). Low-concentration capsaicin promotes colorectal cancer metastasis by triggering ROS production and modulating Akt/mTOR and STAT-3 pathways. Neoplasma.

[114] Qin J.C., Yu W.T., Li H.X., Liang Y.Q., Nong F.F., Wen B. (2021). Cold exposure and capsaicin promote 1,2-dimethylhyrazine-induced colon carcinogenesis in rats correlates with extracellular matrix remodeling. World J. Gastroenterol..

[115] Majumdar A.P., Banerjee S., Nautiyal J., Patel B.B., Patel V., Du J., Yu Y., Elliott A.A., Levi E., Sarkar F.H. (2009). Curcumin synergizes with resveratrol to inhibit colon cancer. Nutr. Cancer.

[116] Manikandan R., Beulaja M., Arulvasu C., Sellamuthu S., Dinesh D., Prabhu D., Babu G., Vaseeharan B., Prabhu N.M. (2012). Synergistic anticancer activity of curcumin and catechin: An in vitro study using human cancer cell lines. Microsc. Res. Tech..

[117] Jin G., Yang Y., Liu K., Zhao J., Chen X., Liu H., Bai R., Li X., Jiang Y., Zhang X. (2017). Combination curcumin and (-)-epigallocatechin-3-gallate inhibits colorectal carcinoma microenvironment-induced angiogenesis by JAK/STAT3/IL-8 pathway. Oncogenesis.

[118] Hu W.H., Chan G.K., Duan R., Wang H.Y., Kong X.P., Dong T.T., Tsim K.W. (2019). Synergy of Ginkgetin and Resveratrol in Suppressing VEGF-Induced Angiogenesis: A Therapy in Treating Colorectal Cancer. Cancers.

[119] Santana-Gálvez J., Villela-Castrejón J., Serna-Saldívar S.O., Cisneros-Zevallos L., Jacobo-Velázquez D.A. (2020). Synergistic Combinations of Curcumin, Sulforaphane, and Dihydrocaffeic Acid against Human Colon Cancer Cells. Int. J. Mol. Sci..

[120] Papi A., Farabegoli F., Iori R., Orlandi M., De Nicola G.R., Bagatta M., Angelino D., Gennari L., Ninfali P. (2013). Vitexin-2-O-xyloside, raphasatin and (-)-epigallocatechin-3-gallate synergistically affect cell growth and apoptosis of colon cancer cells. Food Chem..

[121] Langner E., Lemieszek M.K., Rzeski W. (2019). Lycopene, sulforaphane, quercetin, and curcumin applied together show improved antiproliferative potential in colon cancer cells in vitro. J. Food Biochem..

[122] Chung S.S., Dutta P., Austin D., Wang P., Awad A., Vadgama J.V. (2018). Combination of resveratrol and 5-flurouracil enhanced anti-telomerase activity and apoptosis by inhibiting STAT3 and Akt signaling pathways in human colorectal cancer cells. Oncotarget.

[123] He W.T., Zhu Y.H., Zhang T., Abulimiti P., Zeng F.Y., Zhang L.P., Luo L.J., Xie X.M., Zhang H.L. (2019). Curcumin Reverses 5-Fluorouracil Resistance by Promoting Human Colon Cancer HCT-8/5-FU Cell Apoptosis and Down-regulating Heat Shock Protein 27 and P-Glycoprotein. Chin. J. Integr. Med..

[124] La X., Zhang L., Li Z., Li H., Yang Y. (2019). (-)-Epigallocatechin Gallate (EGCG) Enhances the Sensitivity of Colorectal Cancer Cells to 5-FU by Inhibiting GRP78/NF-κB/miR-155-5p/MDR1 Pathway. J. Agric. Food Chem..

[125] Yang S., Li W., Sun H., Wu B., Ji F., Sun T., Chang H., Shen P., Wang Y., Zhou D. (2015). Resveratrol elicits anti-colorectal cancer effect by activating miR-34c-KITLG in vitro and in vivo. BMC Cancer.

[126] Donald E.L., Stojanovska L., Apostolopoulos V., Nurgali K. (2017). Resveratrol alleviates oxidative damage in enteric neurons and associated gastrointestinal dysfunction caused by chemotherapeutic agent oxaliplatin. Maturitas.

[127] Yin J., Wang L., Wang Y., Shen H., Wang X., Wu L. (2019). Curcumin reverses oxaliplatin resistance in human colorectal cancer via regulation of TGF-β/Smad2/3 signaling pathway. OncoTargets Ther..

[128] Cao P., Xia Y., He W., Zhang T., Hong L., Zheng P., Shen X., Liang G., Cui R., Zou P. (2019). Enhancement of oxaliplatin-induced colon cancer cell apoptosis by alantolactone, a natural product inducer of ROS. Int. J. Biol. Sci..

[129] Wu W., Dong J., Gou H., Geng R., Yang X., Chen D., Xiang B., Zhang Z., Ren S., Chen L. (2021). EGCG synergizes the therapeutic effect of irinotecan through enhanced DNA damage in human colorectal cancer cells. J. Cell. Mol. Med..

[130] Su P., Yang Y., Wang G., Chen X., Ju Y. (2018). Curcumin attenuates resistance to irinotecan via induction of apoptosis of cancer stem cells in chemoresistant colon cancer cells. Int. J. Oncol..

[131] Zhang C., Xu Y., Wang H., Li G., Yan H., Fei Z., Xu Y., Li W. (2018). Curcumin reverses irinotecan resistance in colon cancer cell by regulation of epithelial-mesenchymal transition. Anti-Cancer Drugs.

[132] Borah G., Bharali M.K. (2021). Green tea catechins in combination with irinotecan attenuates tumorigenesis and treatment-associated toxicity in an inflammation-associated colon cancer mice model. J. Egypt. Natl. Cancer Inst..

